# Uncovering the building blocks of rural entrepreneurship: A comprehensive framework for mapping the components of rural entrepreneurial ecosystems

**DOI:** 10.1016/j.heliyon.2024.e24139

**Published:** 2024-01-06

**Authors:** Brilliant Asmit, Togar Mangihut Simatupang, Bambang Rudito, Santi Novani

**Affiliations:** a Institut Teknologi Bandung, Jalan Ganesha 10, Bandung, 40132, Indonesia; b Universitas Riau, Kampus Bina Widya KM 12.5, Pekanbaru, 28293, Indonesia

**Keywords:** Entrepreneurial ecosystem, Rural entrepreneurship, Environmental resources, Co-creation, Local economic potential, Regional development, Citation analysis, Co-occurrence analysis

## Abstract

Entrepreneurship is a crucial driver of economic growth, especially in rural areas. Understanding the unique components that contribute to the success of rural entrepreneurial ecosystems is essential. This study presents a cutting-edge approach to uncover the essential components of rural entrepreneurial ecosystems that support rural entrepreneurship. We employ bibliometric techniques that enable us to leverage the Scopus database's academic paper metadata related to entrepreneurial ecosystems. Through citation analysis, we identify a core network of studies related to entrepreneurial ecosystems. Co-occurrence analysis visualizes the clusters of the most prominent components in both general and rural entrepreneurial ecosystems. We interpret the results based on a literature review. Our study categorizes the essential components into two: actor (academics, business, government, and community) and non-actor (human capital, network, entrepreneurial culture, financial systems, governance systems, infrastructure, environmental resources, and market) components. Environmental resources are critical in distinguishing the conditions of an entrepreneurial ecosystem in rural areas. This component represents the uniqueness and local economic potential of smaller areas, whereas in general entrepreneurial ecosystem studies, this component might not be a concern. By uncovering the components of rural entrepreneurial ecosystems, our study provides insights that can help policymakers, practitioners, and academics better support rural entrepreneurship and promote economic growth in rural areas.

## 1 Introduction

Rural areas have the potential to play a significant role in the development of regional economies as they offer valuable natural resources, such as agriculture, and are attractive tourism destinations [1]. Developing countries can leverage rural areas as a foundation for regional economic growth [2]. However, despite their abundance of natural resources, rural areas typically face limitations in other critical factors of production, notably labor and capital [3]. These limitations represent a gap that hinders the advancement of rural economies. One way to fill this gap is through the fourth factor of production, which is entrepreneurship [4].

Rural areas require space to grow and develop their economies, and rural entrepreneurship can fill this gap to accelerate the rural economy [5]. As main actors, rural entrepreneurs face various challenges when creating and building new ventures. These challenges include limited facilities and infrastructure, limited access to resources and markets, undeveloped social demands, minimal exposure to funding opportunities, a lack of human capital, and less supportive business networks. Most underdeveloped areas face these issues [6]. The pivotal role of entrepreneurship becomes apparent when its inherent capacity to identify and seize promising business opportunities is considered [7]. Exposing a community to entrepreneurial education increases its ability to exploit novel businesses [8], and these entrepreneurial activities accelerate economic growth [9] and sustainable development [10]. Although they are often applied in broader regional contexts [11], the foundational concepts of entrepreneurship are equally pertinent in rural settings. Thus, entrepreneurship has emerged as a powerful force propelling rural areas toward economic prosperity and sustainable development.

Entrepreneurship studies have expanded in scope to the personal, organizational, and regional levels. Regional economics and theories under this economic sub-discipline can relate to entrepreneurship studies, such as economic growth and regional development theories. Marshall's [12] industrial district theory shows that regional milieu affects regional economic development. A region consists of social and economic relations that affect the productivity of local firms [13]. Compared to Marshallian studies, Schumpeterian studies show that regional conditions affect local entrepreneurship [14]. These relations forge a system of multiple actors aiming to achieve economic development. The actor–network theory [15] is closely related to the interaction of multiple actors within a network to determine the collective output. Therefore, to study regional entrepreneurship, it is necessary to focus on actors or organizations other than business entities, such as higher education institutions, communities, and governments. In line with actor-network theory, collective action theory [16] discusses the common interests of actors and how they collaborate to achieve common goals. Although the main goals of each do not focus on entrepreneurship, they are at least interested in regional economic development. These actors have individual purposes, but collectively they have common purposes within their ecosystems.

The term entrepreneurial ecosystem (EE) was first introduced in academic literature by Cohen in 2006 [17]. Previously, it was referred to as the entrepreneurial or entrepreneurship system [18]. The concept of the entrepreneurship ecosystem gained traction among scholars in the 2000s, partly driven by the rise of Silicon Valley as a successful regional entrepreneurship [19]. An EE is a complex system [20] that adapts to support entrepreneurial performance [21]. We consider EE as a foundational structure that supports entrepreneurial activities in specific geographical regions. This structure comprises actor and non-actor components that act synergistically as catalysts for generating entrepreneurial activities as output.

Numerous studies employing the EE framework have focused on the national level [22] or have delved deeply into specific industries [23]. However, when considering more constrained geographic scopes such as rural areas, the composition of the EE differs from the broader norm. Aguilar [24] and McKague [25] strengthened this by stating the essential conditions to consider in a rural EE, including poverty, access to technology and financing, and cultural values. However, as stated earlier, the structure of an EE does not consist of the regions' values but also the actors’ existence to make the EE work as a social system.

A small gap exists in addressing the fact that some crucial values have been excluded from existing rural EE studies. Therefore, the current literature requires a holistic examination using a bibliometric approach, so as not to ignore the critical principles of EE in general, to identify the essential components of less densely populated regions. Our study aims to delineate and ascertain the essential components of a rural EE and contribute to enhancing rural entrepreneurship by investigating and understanding these components.

Section 2 provides a comprehensive overview of the materials, methodology, and research design of this study. This is followed by a discussion of the findings in Section 3. Finally, in Section 4, we draw conclusions based on the analysis and outcomes presented.

## 2 Materials and methods

**Fig. 1:**
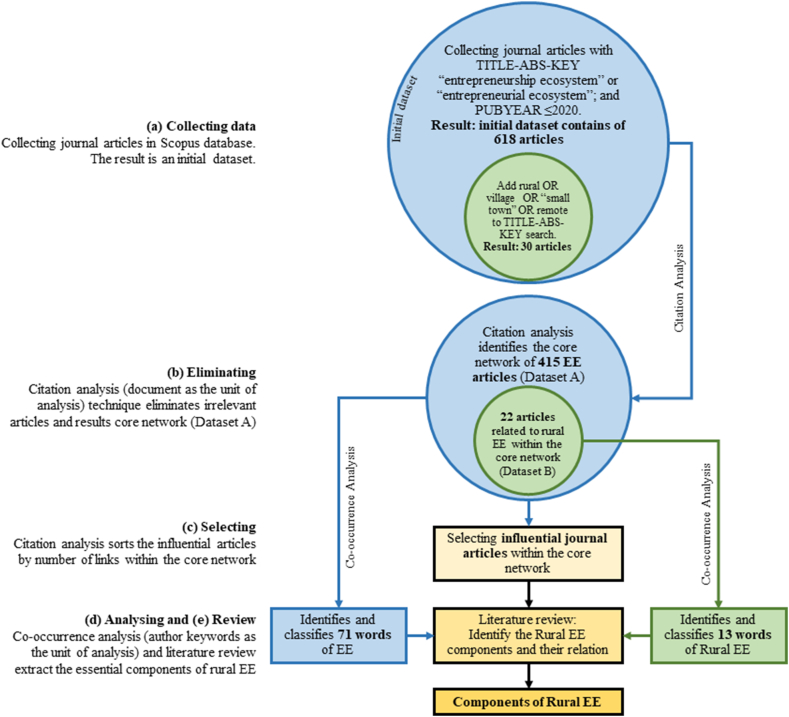
Methodological framework.

The use of bibliometrics as a literature review method is not new in entrepreneurship studies. Scholars have applied this method in various ways to achieve specific goals and to offer unique insights. For instance, Chandra [26] combined topic mapping, co-citation, and overlay visualization to identify the emergence of entrepreneurship study topics, whereas Theodoraki et al. [27] employed co-occurrence, co-authorship, bibliographic coupling, and co-citation techniques to examine a holistic perspective for building sustainable EE. As illustrated in Fig. 1, the purpose of this study is to apply two popular bibliometric techniques: citation and co-occurrence analysis. We used citation analysis to analyze the initial dataset, filter it, and finalize it (Fig. 1b and c). We validated a dataset consisting of relevant articles. The follow-up investigation used a co-occurrence analysis of author keywords (Fig. 1d), combined with a literature review (Fig. 1e), to identify the essential components of a rural EE.

The analysis relied on network visualization and a computational approach. Therefore, this study used VOSviewer software, which visualizes the bibliometric network based on the similarities of publication metadata, such as author keywords, author names, and citations [28].

### 2.1 Data collection

In the initial stage, a suitable dataset was compiled by establishing a protocol for setting search restrictions and eliminating irrelevant data. The Scopus database was used as a data source. Only published journal articles were included because published knowledge has been robustly peer-reviewed. Moreover, this type of literature provides a consistent structure of information, such as titles, keywords, references, and citations.

As EE is the central topic of this study, we used the search keyword “entrepreneurial ecosystem”; other researchers have also used “entrepreneurship ecosystem.” Therefore, the data collection phase started with the keyword search for “entrepreneurial ecosystem” OR “entrepreneurship ecosystem” on Title-Abstract-Keyword. The search was conducted in December 2021. We limited our search to the latest publications, published in 2020. The search yielded 621 articles with publication years ranging from 2006 to 2020. At this stage (Fig. 1a), we obtained an initial dataset containing 618 articles after removing duplicates. In addition, we used rural words as search combinations to distinguish between rural articles. Previous studies have mentioned other terms for rural areas, namely villages [29], small towns [30], and remote areas [31], along with EE. Therefore, we used keyword combinations of “entrepreneurial ecosystem,” and “entrepreneurship ecosystem,” as well as “village,” “small town,” or “remote area” to yield a dataset containing 30 articles.

### 2.2 Data analysis

#### 2.2.1 Citation analysis

The initial dataset contained irrelevant articles even though they contained the keyword “entrepreneurial ecosystem.” Citation analysis can distinguish relevant articles from the rest, which may be missed if the articles are read as usual. According to Klavans and Boyack [32], direct citations accurately depict the knowledge taxonomy. Therefore, citation analysis is a convenient first step for screening valid inputs for further analysis.

**Fig. 2:**
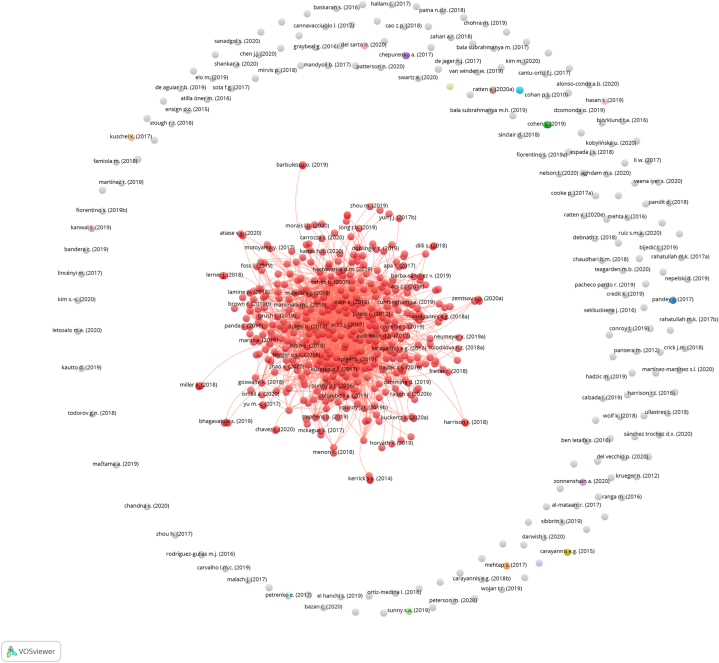
Visualization of citation analysis in 618 articles in EE studies, 415 of them (red label) form the core network (processed using VOSviewer, online version: https://tinyurl.com/yrhbarmf)

The collection of articles connected by citation links formed the core network (Fig. 2), which established Dataset A. Subsequently, articles on rural areas included in the core network formed Dataset B.

#### 2.2.2 Co-occurrence analysis

Co-occurrence analysis uses author keywords as the analysis unit to delineate EE components. Co-occurrence analysis visualizes the relationships between words. Moreover, it depicts the proximity of words based on their frequency of appearance with other words [28]. Words close to each other formed clusters, as shown in Fig. 5 (Dataset A) and 6 (Dataset B).

Bias in interpreting words can occur because of nonstandard word forms. Therefore, it was essential to tidy the appearance of the keywords. The first step was to standardize the words using American English expressions (e.g., use “behavior” instead of “behavior”), followed by addressing plural words (e.g., “entrepreneurs” to “entrepreneur”), abbreviations (use “global entrepreneurship index” instead of “GEI”), literature classification codes (e.g., JEL classification), and variety of phrases with the same meaning (e.g., “entrepreneurial ecosystems” and “entrepreneurship ecosystem”). These stipulations produced a thesaurus to tidy up keywords. Dataset A yielded 544 keywords. However, not all keywords were analyzed because they created map visualization clutters. Therefore, we only processed keywords with at least five occurrences, yielding 79. Eight further words were excluded, including search keywords at the data collection stage, namely “entrepreneurial ecosystem,” “entrepreneurship,” and “ecosystem,” which are the core topics. Apart from that, we excluded words unrelated to EE components, namely location names (e.g., “china”), research and analysis related (e.g., “literature review” and “global entrepreneurship monitor”), and other irrelevant terms (e.g., “COVID-19” and “crisis”). The resulting map robustly visualizes the 71 terms in EE studies (Fig. 5). Dataset B contained 60 keywords. We excluded ten words with the same criteria in processing the previous dataset (namely “ecosystem,” “entrepreneurship,” “entrepreneurship model,” “literature review,” “forecast,” “global entrepreneurship monitor,” “bangladesh,” “chile,” and “mexico”). Unlike Dataset A, the analysis of Dataset B included the term “entrepreneurial ecosystem” because the number of inputs was smaller than Dataset A. In addition, we used the word “rural” to visualize the relationship between words in a more extensive network without being separated. Finally, the analysis included only words with two occurrences as the threshold and created a network map of 16 keywords in rural EE studies (Fig. 6).

#### 2.2.3 Systematic literature review and network analysis

Bibliometrics is a valuable method; however, it has limitations in identifying the essential components of EE. A limitation is that the analysis technique relies on registered article metadata, which is vulnerable to missing critical information that is not explicitly mentioned as metadata. Therefore, interpreting the results requires a qualitative approach to review the article and understand the entire idea of the network.

**Fig. 3:**
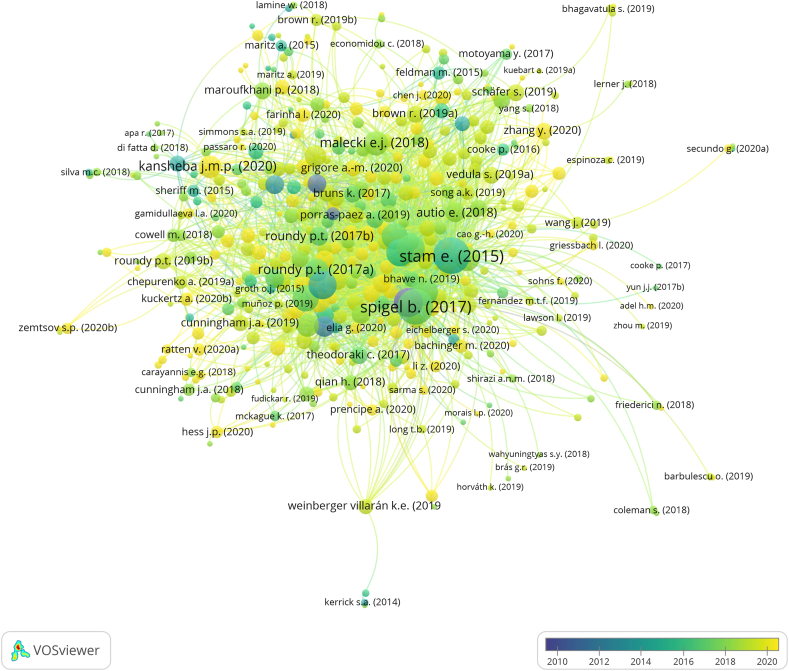
Visualization of the number of links (label size) and the publication year (label color) of the EE studies core network (processed using VOSviewer, online version: https://tinyurl.com/23hq9lku)

We utilized the citation (Fig. 3) and co-occurrence networks (Fig. 5, Fig. 6) as essential information for reviewing the observed terms in influential articles. At this stage, every word in the spotlight was traced through articles related to the citation network. Thus, we interpreted the relationship between words from the co-occurrence network based on the article review results. This series of analyses will answer recent study questions.

## 3 Result and discussion

### 3.1 The core of influential entrepreneurial ecosystem studies: citation analysis

The first objective of citation analysis is to select articles relevant to EE. This stage resulted in 415 articles called the “core network” of EE studies (Fig. 2). Although 203 articles outside this network were also related to EE, they contributed less to this study than to the core. As a result of direct citation analysis, the core network provided an accurate knowledge database for further investigation.

Citation analysis also provides information on the number of links in each document. In citation analysis, the number of links indicates how many times an item (article) is “cited by” other items and references other items (“citing”). Therefore, there are two possible ways to interpret the number of links to an article according to the year of publication (Table 1). First, the earliest published paper shows its impact within the network because the number of links represents the number of “cited by.” Cohen's [17] article on sustainable EE is the earliest and has 88 links, showing how many papers have cited Cohen's articles within the network. Second, a later article with many links gains knowledge by investigating previously published articles. Kansheba and Wald [33]'s is the latest publication with the most links between them. Their paper is a systematic literature review citing 41 articles from the core network. Similar to earlier publications, the later article contributes to the elaboration of EE.

**Table 1:** Top 20 papers with the most links in the core network.

No	Article	Author keywords	Number of links	Cited by^a^
1	Stam (2015) [34]	policy; region	162	563
2	Spigel (2017) [35]	[no author keywords]	150	576
3	Cohen (2006) [17]	community; sustainable	88	297
4	Brown and Mason (2017) [36]	policy; growth	77	221
5	Alvedalen and Boschma (201) [37]	cluster; network	77	214
6	Autio et al. (2014) [38]	innovation; policy; innovation	75	536
7	Mack and Mayer (2016) [39]	economic development; evolutionary; geography; policy	70	204
8	Acs et al. (2017) [40]	cluster; governance; unicorn	67	279
9	Audretsch and Belitski (2017) [41]	city; start-up	63	245
10	Spigel and Harrison (2018) [42]	cluster; culture; network; regional innovation; region	54	206
11	Malecki (2018) [43]	[no author keywords]	51	168
12	Roundy, Bradshaw, and Brockman (2018) [44]	new venture; resilience; start-up	42	106
13	Roundy, Brockman, and Bradshaw (2017) [45]	complexity; new venture; start-up	41	140
14	Kansheba and Wald (2020) [33]	antecedent; entrepreneur; outcome; start-up	41	4
15	Cavallo, Ghezzi, and Balocco (2019) [46]	new venture; system	38	116
16	Pitelis (2012) [47]	[no author keywords]	32	124
17	Roundy (2020) [48]	[no author keywords]	32	3
18	Autio et al. (2018) [49]	knowledge; business model; digital; spatial; start-up	29	278
19	Roundy (2017) [30]	community; new venture; regional development; urban	27	48
20	Nambisan and Baron (2013) [50]	[no author keywords]	26	247

a Records per December 2021.

Theodoraki et al. [27] divided the evolution of EE publications into three categories: emergence (2006–2014), approval (2014–2017), and expansion (2017 and later). The top three articles with the most links and those considered influential represented each period: Cohen [17], Stam [34], and Spigel [35]. The visualization (Fig. 3) shows no single citation link within them; however, these articles significantly contributed to the core network.

Stam is quite popular in EE studies because his model presents the entrepreneurial ecosystem's framework, systemic conditions, outputs, and outcomes. The Stam model inspired others to study actors' networks [51], regional entrepreneurship [52], and non-urban EE [30]. Spigel [35] was the most prominent article cited in EE and entrepreneurship studies [27]. This study classified EE into three categories of attributes: cultural, social, and material. Cohen [17] was the earliest publication in the core network that introduced the concept of EE. Cohen [17] stressed the importance of interconnected actors in certain areas for supporting and facilitating new ventures.

A citation analysis of the core network identified influential articles with fundamental concepts of EE. These concepts have influenced other scholars’ investigations of EE, including its components and outcomes [34]. These components can be categorized as actors [17,43] or non-actors, such as conditions, attributes, and shared values [35,45]. Early scholars have studied EE in different areas and identified its essential components. The different conditions of an area vary the composition of ecosystem components. This study does not ignore these findings and serves as a knowledge base for explaining the specific setting of rural EE in the following co-occurrence analysis.

### 3.2 Terms in the entrepreneurial ecosystem in general and rural context: co-occurrence analysis

**Fig. 4:**
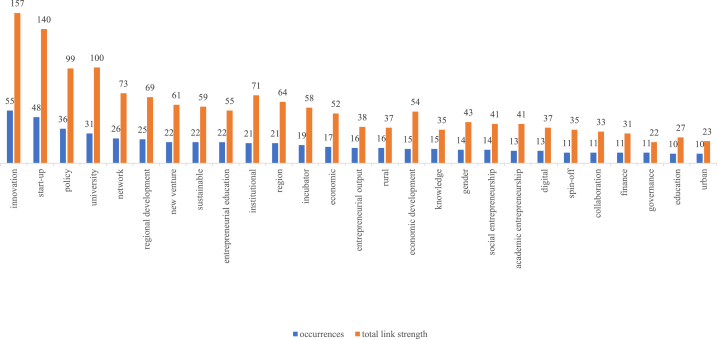
Keywords that occur in the core network of EE publications.

**Fig. 5:**
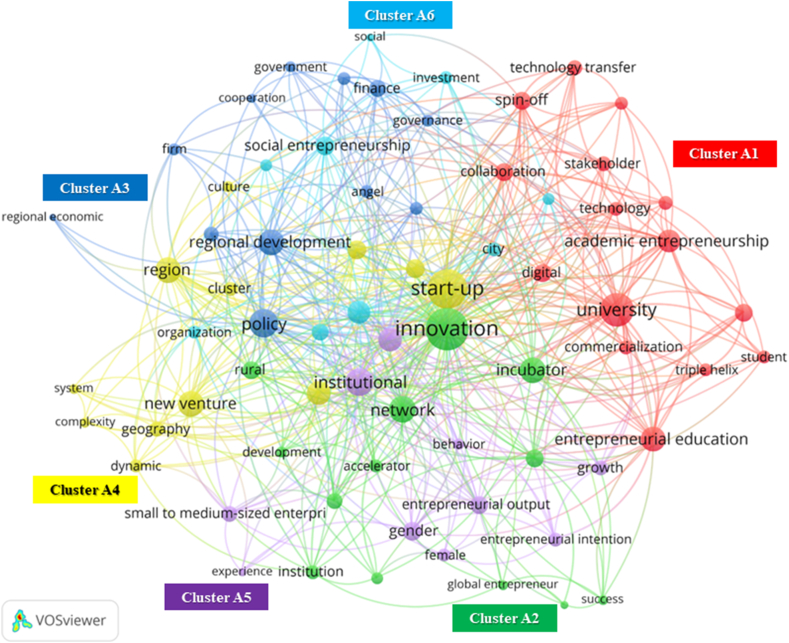
Visualization of clusters (label color) and total link strength (label size) of author keywords in EE studies (processed using VOSviewer, online version: https://tinyurl.com/26ja39rk)

Co-occurrence analysis provides popular words based on their occurrence in the core network, as displayed in Fig. 4. The total link strength in the diagram represents the number of times words in an article are cited by other authors. Based on the influential articles, 71 words can be categorized as actor components, non-actor components, outputs, and outcomes (Table 2). The first category is related to actors: “university,” “triple helix,” “stakeholder,” and “community” [43,53,54]. The non-actor components include “policy,” “network,” “institutional,” “collaboration,” “education,” “culture,” and “geography” [35,55]. In addition, we identified terms in other categories, namely, the output and outcome of EE. The output of the EE category includes “innovation,” “start-up,” and “new venture” [56,57]. The outcome category consists of “sustainable,” “regional,” and “economic development” [34,35,58]. The core network illustrates these terms in six clusters (Fig. 5). Next, we extracted the essential components of EE using cluster information and categorizing terms. Terms based on clusters and categories (Table 2) help analyze the critical features and their interrelationships that support EE.

**Fig. 6:**
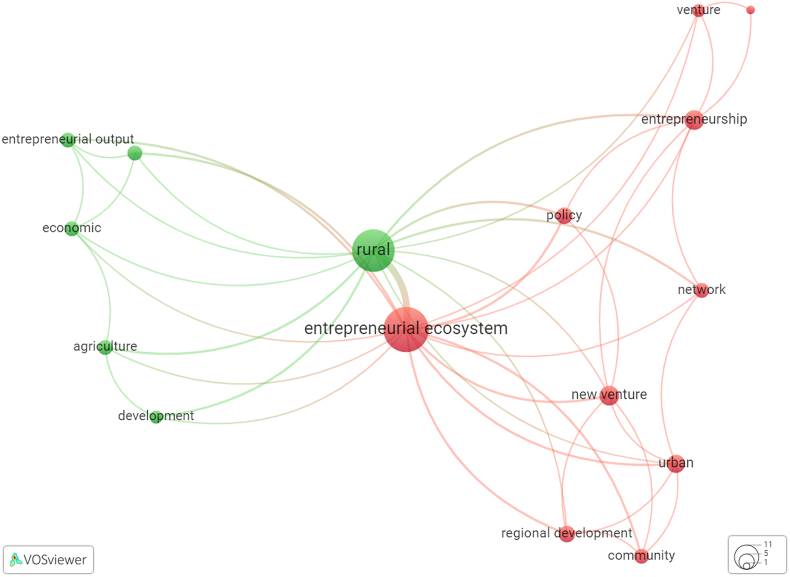
Visualization of words total link strength (label size) in rural EE studies (processed using VOSviewer, online version: https://tinyurl.com/2dk6waqj).

**Table 2:** List and category of words regarding the co-occurrence analysis within EE studies core network.

Cluster	Word and Total Link Strength				
	Actor Component	Non-actor Component	Output	Outcome	Area Context
A1	university (66) stakeholder (16) student (13) triple helix (13)	entrepreneurial education (38) collaboration (22) digital (21) technology transfer (16) technology (15) research and development (13)	academic entrepreneurship (34) spin-off (23) entrepreneurial university (22) commercialization (15)		global (14)
A2	incubator (41) institution (15) accelerator (12)	network (44) education (22) support (18) social capital (11)	innovation (97) global entrepreneur (8) success (8) entrepreneur (6)	development (10)	rural (21)
A3	angel (12) government (10)	policy (50) finance (19) venture capital (13) governance (11) cooperation (7)	business model (12) firm (11)	regional development (41) regional economic (4)	urban (16)
A4		knowledge (24) geography (16) dynamic (13) culture (9) complexity (8) system (8)	start-up (86) new venture (41) regional innovation (24) cluster (15)	economic development (36)	region (42)
A5	female (12)	institutional (48) gender (25) behavior (9)	entrepreneurial output (22) small to medium-sized enterprise (18) growth (16) entrepreneurial intention (14) experience (6)	economic (36)	
A6	community (20) organization (13)	investment (13) information and communication technology (11) social (6)	social entrepreneurship (24) social enterprise (11)	sustainable (33)	city (15)

The co-occurrence analysis of Dataset B visualized the relationship between terms in the rural EE study (Fig. 6). From the 22 publications on EE in a rural context, the co-occurrence analysis of the keywords revealed 13 terms by excluding the words “rural” and “entrepreneurial ecosystem” (Table 3). Prominent words in this small network were “community,” “policy,” “network,” and “new venture,” which also appeared in the network of EE studies.

**Table 3:** List and category of words (with total link strength) regarding the co-occurrence analysis within rural EE studies.

Actor Components	Non-Actor Components	Output	Outcome
community (5) institution (5) venture (4)	policy (6) network (5) agriculture (5)	new venture (8) entrepreneurship (8) entrepreneurial output (5)	regional development (6) economic (5) development (4) sustainable (2)

“Community” belongs to the actor category that often occurs with “institution.” Scholars have highlighted the roles of these two actors in EE, giving birth to new ventures [31] and regional development as outcomes [59]. Development has the most links within the network, which means that EE in a rural context extensively studies regional development. We conclude that EE in a rural context was investigated to pursue the development of an area. This network also highlights regional characteristics apart from the community in the actor category, namely agriculture in the non-actor category. This is a concern for discussion, because, according to Freitas and Kitson [31], actors adapt to ecosystem conditions.

Cluster A1 includes studies on EE involving academia as an influencing actor. Universities orchestrate an EE actors’ network [60]. Restricting university influence affects a regional EE performance in terms of knowledge and technology transfer [61]. Other higher education outputs include academic entrepreneurship and knowledge commercialization [62], which require collaboration with other actors to boost output, such as university spin-offs [63].

Innovation frequently co-occurs with universities (link strength of six between them), start-ups (six), and new ventures (five). In Cluster A2, innovation has the most links (five) with the incubator. The proximity and network of actors and resources are critical success factors for incubation, as incubators pass on knowledge from universities to incubated participants [64]. This contribution is in line with the expected output of EE, which encourages the creation of innovative ventures [38]. Another support form, the accelerator, contributes to the scaling-up of ventures [65], and the outcome is the development of an ecosystem [66].

Rural areas have a total link strength of 21, often appearing in policies, developments, and networks. Cowell [67] stated that EE requires a network. Each type of ecosystem has a different capability for providing this luxury. Freitas and Kitson [31] compared EEs of a core region, Catalonia, and a remote area, the Canary Islands, Spain. They concluded that remote-area ventures perceive a lower supporting ecosystem than core-region ventures, as the latter has institutions that facilitate mutually beneficial networks.

The formation of new firms is an EE's performance measure, and reciprocally, firms are important in nurturing entrepreneurial activity within an ecosystem [68]. Establishing a business is the output of the interactions between the components of an EE. Cluster A3 shows the components of financing matters (finance and capital). For instance, Ghio et al. [69] argued that local banks, as the source of financing, determine the formation of high-tech ventures. Moreover, according to Zhang and Roelfsema [70], Finance also supports the business scale-up. Interestingly, both publications [69,70] mention financial relationships with culture, such as residents' attitudes toward trusting local cooperative banks.

EEs require abundant knowledge to empower actors in facilitating opportunity discovery. Opportunity-related knowledge can come from other actors in a cluster, such as established local ventures that share business experiences [49], or from outside the cluster, such as university knowledge [43]. In smaller regions such as rural areas, the expected output of opportunity discovery is the establishment of new local ventures. Knowledge enables locals to understand regional potential and pursue business opportunities courageously. Cetindamar et al. [71] explained that EE actor collaboration is one way to increase knowledge.

An EE is a system consisting of interdependent actors in which the interaction between them involves social values and culture. Spigel's [35] placed cultural attributes as fundamental elements of EE. Culture can support entrepreneurship and positively support the creation of ventures; otherwise, it hinders it [72]. Culture has always been a critical component of EE; however, an ecosystem requires a dominant entrepreneurial culture to support entrepreneurial activities.

Experience is linked to networks, small-to medium-sized enterprises, and new ventures. Actors' prior experience supports entrepreneurial activities within the network, including increasing venture performance [73] and value co-creation [74]. The captured experience does not originate from a single actor but from all the actors involved. Actors collectively accumulate experience and develop human capital to perform entrepreneurship activities [75]. In rural areas, developed human capital means enabling rural communities to identify business opportunities and problem-solving cleverly. Therefore, rural people rely on network quality to build their knowledge and experience. For an ecosystem, the actor's prior experience contributes to the development of the EE component [76]; as in rural areas, it will undoubtedly give birth to supporting policies and developing local resource management.

Community and social issues are prominent in Cluster A6. The value chain of entrepreneurial activity output involves communities [77]. A community shares valuable local wisdom, generates ideas, and promotes local products. Therefore, other EE actors must focus on building the capacity of entrepreneurial communities to support and revitalize an ecosystem [78]. Studies on rural EE link communities to regional development and new ventures (Fig. 6). These unique resources form the basis for rural areas to build entrepreneurship. Local communities are the closest actor in taking advantage of this unique opportunity. The development of areas in which communities participate reflects the benefits of the site, such as tourism [79] and agriculture [80].

### 3.3 The proposed components of the rural entrepreneurial ecosystem

The previous section discussed how the components of EE are interrelated and correspond to outputs and outcomes, both generally and specifically, in rural areas. Thus, we have a rationale for asserting that the terms in the component category (actors and non-actors) are essential components of rural EE. However, the list contained similar terms; therefore, we grouped it into standard terms corresponding to its description. For instance, “university,” “student,” and “incubator” were grouped as “academic,” describing this component as actors contributing to knowledge transfer. This agglomeration is summarized in Table 4.

**Table 4:** Essential components of a rural EE.

Components	Related words in co-occurrence analysis	Description
**Actor components**		
Academic	university; student; incubator; accelerator	This group includes universities, research institutes, and incubators, contributing knowledge transfer to the business and community group.
Business	angel; entrepreneur; small to medium enterprise; firm; enterprise; venture	Besides the entrepreneur, this group comprises a cooperative, business consultants, incubator, CSR, supplier, distributor, and financial institution.
Government	government; institution.	The policymaker acts as a regulator and has an important role in developing their region.
Community	community; stakeholder; organization	The society within the ecosystem (locals) share the culture and demands (market).
**Non-actor components**		
Human capital	entrepreneurial education; education; technology transfer; knowledge; research and development	Knowledgeable and experienced talent in rural areas creatively generate and commercialize the business idea. The connectivity of diverse actors enables knowledge sharing and exchange.
Network	collaboration; network; support; cooperation; institutional; complexity; dynamic	The business density of an area contains incubators, advisors, mentors, professional services, customers, entrepreneurs, and peers that support the entrepreneurship process.
Entrepreneurial culture	culture; behavior; social	The shared entrepreneurial values and behavior within an ecosystem.
Financial systems	finance; venture capital; investment	The capability of an ecosystem to provide funding. It comes from friends and family, private equity, angel investors, financial institutions, and debt access.
Governance systems	policy; governance; system; institutional	Policies support business creation and growth—government and regulation support resource exploitation, which aims for rural economic development.
Infrastructure	digital; technology; information and communication technology	Infrastructure supports the exploitation of opportunities and business activities. Basic infrastructure connecting the resources, production units, and markets.
Environmental resources	geography; agriculture	A rural area has different natural capital. As a comparative advantage, villagers can benefit from the natural resources to produce their area's specific product.
Market		Demands within an ecosystem area and the accessible external market.

#### 3.3.1 The contributing actors

EEs involve multiple actors with various roles in Ref. [36]a complex system [36]. Purbasari et al. [54] categorized the actors of EE into market, professional, government, finance, society, and entrepreneurs. All of these interact with each other in the network. In a network, actors exchange knowledge and collaborate to adapt to a dynamic ecosystem.

As leading actors and a part of the business community, entrepreneurs distinguish between small-medium enterprises (SMEs) and innovation-driven enterprises [67]. Each has different needs; for instance, SMEs depend on government regulation to extend their existence and maintain equilibrium. In contrast, innovation-driven enterprises need to connect with angel investors to continuously scale up. Therefore, business actors should be in the center of the ecosystem and be relevant to other actors.

Academic infiltration enables knowledge spillover within ecosystems [81]. This knowledge can improve society's intelligence in executing opportunities to create commercial ventures [82]. Universities offer multidisciplinary knowledge related to knowledge spillover that helps shift people's intention to entrepreneurial activities [83]. Knowledge within an ecosystem enables co-creation [17], which recognizes opportunities for exposure to markets and resources. Kumar and Das [84] asserted that academic institutions construct entrepreneurial intentions. Academics play a vital role in spreading knowledge through disseminating research results and community services, which connect the rural entrepreneur to other components to recognize and exploit business opportunities. Therefore, this study proposes that academia contributes to rural entrepreneurship.

Thompson et al. [85] argued that EE is formed by a bottom-up process rather than a top-down approach. If a strategic approach is needed to develop an ecosystem [86], EE requires strategic actors, such as policymakers [87]. This ecosystem is already present, and it develops and reshapes its form along with its components. Zahedi and Otterpohl [88] argued that actors in EE contribute to shaping ecosystems. In an ecosystem, the government plays a vital role in the development of vision. The governing strategy is to mix and match EE components, such as supportive regulations, to expose the market and resources available to rural societies [89]. Moreover, it can work well with good leadership performance [90]. In rural areas, local governments are relevant to regional development by enabling actor networks to collaborate and manage resources.

Reciprocally, EE helps actors perform effectively in running an institution's business [20,91]. EE provides the services that actors require to perform entrepreneurial activities. In response, the ecosystem asks actors to contribute to the development of entrepreneurship in the area. This finding suggests that mutual relationships exist between EE and actors, indicating a collaborative growth dynamic. Actors have room to improve, grow, develop, and reshape EE, increasing their services to entrepreneurial performance within the area. We classified actors into four groups: *academic, business, community,* and *government*. The quadruple helix [53] underlies the classification of these actors and the results show the same grouping. Moreover, this grouping makes it easier to communicate this study because the quadruple helix is familiar to academics such as Barbulescu and Constantin [92] and Schütz et al. [93].

#### 3.3.2 Essential components of rural entrepreneurial ecosystem

Actors’ interactions within rural areas affect the ecosystem structure that supports entrepreneurship, and their manifestation appears in changes in the business environment. Conversely, this environment assists actors in the success of rural entrepreneurship. Non-actor components include *human capital, network, entrepreneurial culture, financial systems, governance systems, infrastructure, environmental resources, and market*.

***Human capital***. This component is associated with knowledge, experience, skills, workforce, and talent [94]. Human capital is a decisive factor in entrepreneurial success. Education and prior experience define an entrepreneur's ability to achieve business goals [75]. Knowledge spillover can increase the exploitation of business opportunities to create and produce more [[95], [96], [97]]. The more knowledge spills within an ecosystem, the more it contributes to entrepreneurial behavior and the intention to start and manage a business [84,98]. The intellect of rural societies can be improved by propagating knowledge across regions. Consequently, rural communities can recognize opportunities associated with market consciousness and benefit from local resources.

Human capital becomes the bedrock upon which rural entrepreneurship is constructed. Its role in driving innovation, stimulating entrepreneurial intentions, and optimizing resource utilization underscores its indispensable status in rural EE.

***Entrepreneurial culture***. Entrepreneurial culture plays a role in shaping entrepreneurial knowledge and experience. Spigel [35] placed culture as the base for entrepreneurial activity within an ecosystem. This supports the material and social attributes. By contrast, a well-established culture could also prevent entrepreneurial activities from growing [86]. For instance, old paradigms or norms prevent new, contradictory entrepreneurial values from being implemented in a community.

Regarding the development of EE, a specific culture has been suggested as a component of entrepreneurial culture. This component is associated with the entrepreneurs' shared values in the entrepreneurial environment. Shared values represent community members’ entrepreneurial identities, experiences, and dynamic capabilities [99]. In line with Audretsch et al. [100], local subculture plays a role in the formation of new businesses in their area; essentially fostering an entrepreneurial culture. Community members, as agents attached to new entrepreneurial knowledge and experience, can add value to the established EE. This cycle continuously reshapes and changes entrepreneurial culture over time.

Entrepreneurial culture is a dynamic force within rural EE, continually evolving and reshaping itself through a cyclical process of knowledge sharing, experience accumulation, and the infusion of innovative values. This culture serves as a compass for guiding rural entrepreneurial activities.

***Network***. One type of EE service involves the provision of networks. Supportive institutions, business groups, and farmers’ groups are associated with this component in the rural context. Networks play an important social role in business communities [42,90]. This component enables entrepreneurs to connect business members and the workforce and gain informal knowledge, which is a relevant skill [18]. This network influences the growth of human capital and is a function of academia. Networks also enable entrepreneurs to access funding and potential markets.

Networks provide a dynamic web of connections and resources that not only enhance the capabilities of individual entrepreneurs, but also contribute significantly to the growth and prosperity of rural communities. Networks are critical infrastructures that underpin the success of rural entrepreneurship.

***Governance systems***. As policymaker and regulator, the government plays a significant role in governing ecosystems. Through policy, the government can boost the merits of the system components. According to Cowell et al. [67], SMEs depend on this. A proficient policy can open a network such that business actors can observe and obtain supportive entities, including financing, to exploit business opportunities. Supportive regulations ensure business survival and contribute to long-term viability by offering a sense of certainty regarding market access and resource availability. A well-crafted governance system with rural-business-focused policies creates an ecosystem in which businesses can thrive and play a vital role in rural economic development.

***Financial systems***. The financial component refers to the ecosystem's capability to provide funding [101]. Multiple ways of accessing business funding in rural areas include friends, families, and financial institutions. The latter can provide microcredit to locals. Creating new ventures is clearly one of the funding benefits, and microcredit helps local businesses to grow by adopting advanced technology [102]. Moreover, microfinance contributes to higher living standards in rural areas [103], and provides opportunities to meet new needs.

The financial system in rural EE is far more than a facilitator of funding; it is a catalyst for transformation. Providing access to capital empowers entrepreneurs to bring their ideas to life, fosters economic development, and enhances the well-being of rural communities.

***Infrastructure***. The development of infrastructure in an ecosystem enables the business community to access markets [35]. In addition, infrastructure enables resources to be exploited by the business community, and policy acts as a counterweight to control exploitation to sustain entrepreneurial activities [104]. Infrastructure is more than just physical; it links entrepreneurs to markets and resources, shaping the contours of rural entrepreneurial ecosystems. Its strategic development, guided by thoughtful policies, unlocks business opportunities, ensures resource preservation and fosters a sustainable entrepreneurial landscape. Infrastructure is an essential component through which an entrepreneurial ecosystem can be constructed and thrive.

***Environmental resources***. Environmental resource endowment is essential to EE development [90], particularly in rural areas. However, rural areas have limited access to other resources [105]. This disadvantage can be overcome by utilizing the closest available resources. Natural resources motivate entrepreneurs to start businesses [106] and produce products. Tourism is a business-based environmental resource. Aesthetic and heritage values have become a competitive advantage for blessed rural areas [107,108]. The tourism sector attracts more income from the outside and increases the regional economy.

Environmental resources within rural EE are more than just assets; they are the lifeblood that fuels entrepreneurial activity, fosters distinctive local products, and creates vibrant economic sectors. These resources not only support the ecosystem, but also define the character and potential for sustainable development.

***Market***. Most EE studies do not explicitly place the market in authors’ keywords, titles, or abstracts. The data record only three market occurrences that do not pass the threshold of the number of occurrences. However, upon reviewing influential papers, it is evident that the aforementioned components relate to, and are as important as, the market [35,55] and demand [57] in developing an ecosystem. A region, at the very least, can sustain business as usual with market and resource availability. Therefore, business owners need the certainty of market existence, especially in rural areas where the market is prone to uncertainty. Market certainty is crucial when access infrastructure is questioned in rural areas, because it challenges remoteness [105]. Cunha et al. [106] asserted that better accessibility to the market could contribute to the sustainability of EE. Along with resources, this component requires infrastructure to be exposed to business actors. Moreover, external and local demands encourage productive entrepreneurial activities [109].

The market component within rural EE is not merely a transactional space but a dynamic force that influences and is influenced by various ecosystem elements. Its certainty, accessibility, and alignment with other components collectively shape the entrepreneurial landscape, driving rural areas toward economic fortune.

## 4 Conclusion

Rural entrepreneurial ecosystems (EE) comprise actor and non-actor components. Critical actors within this ecosystem include communities, businesses, academics, and government entities. These actors play a systemic role that reflects and influences various non-actor components, including natural resources, human capital, the market, business networks, entrepreneurial culture, finance, policy, and infrastructure. Previous studies explored how EE components interact systemically to support entrepreneurial activity in specific regions. The conditions and interplay between these components indicate overall ecosystem performance.

Rural EE offers an alternative avenue for developing a rural economy. The components identified within EE contribute to existing literature on rural EE. A more comprehensive understanding of how rural EE supports entrepreneurship is required. To advance our understanding of this area, scholars can extend this study by synthesizing the concept of rural EE with other relevant concepts, such as the entrepreneurial process and co-creation.

EEs aim to nurture entrepreneurship within an ecosystem by driving the entrepreneurship process toward success. The entrepreneurship process begins with entrepreneurs identifying and successfully exploiting business opportunities to create business and innovation. Therefore, EE components must be in good condition for the process to succeed. Regarding this study's results, further research should comprehensively examine the interrelationship between rural EE components and the discovery and exploitation of opportunities in rural areas. Furthermore, this study focuses on reviewing the concept of EE within a rural context, which presents a broad area of investigation. To facilitate a more in-depth analysis, we propose using villages as representative units for further empirical investigation. As an administrative area, villages have institutional structures that would provide a focused perspective on the components of rural EEs.

Additionally, this study identifies the involvement of multiple actors, aligned with the evolving theory of service-dominant logic [110]. This theory introduces the concept of value co-creation, in which various actors exchange value to achieve common objectives. By synthesizing the concepts of value co-creation and EE, it becomes evident that multiple actors in rural EE collaborate to co-create social values, thereby reshaping the ecosystem at the macro level. At the micro level, the ecosystem serves as a platform for co-recognizing and co-exploiting business opportunities. This exploration sparks curiosity and paves the way for a research agenda that delves deeper into how the identified EE components support co-creation in rural entrepreneurship.

Bibliometrics offer many ways to analyze literature, such as selecting various analysis techniques (co-authorship, co-occurrence, bibliographic coupling, and co-citation analysis), data sources (databases), and data types (articles, books, chapters, and conference proceedings). Each method, or a combination of several methods, can provide different outcomes. We acknowledge that this study is limited as it uses only a single database source (Scopus) and data type (journal). It may not encompass all the relevant literature and research on rural entrepreneurial ecosystems. This limitation may have resulted in the exclusion of potentially valuable insights from other sources. There is also an opportunity to study the same context using other data sources. However, further research is required to standardize the metadata from various sources and types. Categorizing components into actor and non-actor categories could oversimplify the complexity of rural entrepreneurial ecosystems. Different areas may exhibit unique characteristics, and this study's approach may not have adequately addressed this heterogeneity. Further research could consider this by narrowing its focus from a general review of rural EE to a more specific examination of one region with the assumption of homogeneity for the components of rural EE.

## Data availability statement

The dataset supporting the findings of this study is openly available at https://doi.org/10.6084/m9.figshare.21640535.

## CRediT authorship contribution statement

**Brilliant Asmit:** Writing – review & editing, Writing – original draft, Visualization, Validation, Software, Resources, Methodology, Formal analysis, Data curation, Conceptualization. **Togar Mangihut Simatupang:** Writing – review & editing, Writing – original draft, Validation, Supervision, Resources, Methodology, Formal analysis, Conceptualization. **Bambang Rudito:** Writing – review & editing, Validation, Supervision, Resources, Data curation. **Santi Novani:** Writing – review & editing, Writing – original draft, Visualization, Validation, Supervision, Resources, Methodology, Data curation.

## Declaration of competing interest

The authors declare that they have no known competing financial interests or personal relationships that could have appeared to influence the work reported in this paper.
